# Advantages of Hypodermic Anesthesia

**Published:** 1911-07

**Authors:** 


### Advantages of Hypodermic Anesthesia.

Lanphear, in the *International Journal of Surgery,* enumerates these as follows:

*Absence of Nausea.*—After operation there is absence of nausea and vomiting—a most important thing, especially in abdominal surgery.

*Abolition of Pain.*—Post-operative pain is conspicuous by its absence. Patients often pass a most comfortable night instead of suffering intensely and sometimes sleep much of the first twenty-four hours succeeding operation, awakening next morning with a demand for food.

*Early Feeding.*—The desire for food may usually be gratified (except, of course, in operation on the stomach and other special work where peristalsis is objectionable), as the secretions are not checked enough to interfere with digestion. This is of great import, particularly when the patient has become weakened before operation and when there has been unusual loss of blood.

*Prevention of Shock.*—When this form of anesthesia is used, shock is absent unless there is excessive hemorrhage, or unless too much ether or chloroform is used. Much of what has been called "shock" in the past was the result of an excessive amount of chloroform and in less degree ether.

*Freedom from Fear.*—A most prominent advantage of this form of anesthesia is the absence of fear or dread of the operation room.

*No Anesthetist Essential.*—A most decided advantage of this form of anesthesia, and especially of true hyoscine-morphine-cactin (H-M-C, Abbott), supplemented by a local anesthetic (Abbott's Anesthaine preferred) when needed, is that a surgeon may perform many operations without the assistance of another doctor. The importance of this in country practice can hardly be overestimated. Curettage of the uterus, repair of cervix, repair of perineum, forceps delivery, operation for strangulated hernia, for appendicitis, reduction of fractures, etc., may be done with this agent, plus a local anesthetic.

*Economy.*—To hospitals, and to many doctors, the cost of ether or even of chloroform, is something of more than passing interest. In hospitals, where many operations are performed, the use of even one tablet an hour or so before the patient is taken to the operation room will reduce the anesthetic bill of the institution one-half or more.

*Lessening Labor.*—To the nurse this form of anesthesia is a God-send. The relief is not only physical, but mental; no anxiety about post-operative pain, vomiting, restlessness, fear and thirst of the patient—all this means much to the conscientious hospital nurse; and the patient who has had both forms of anesthesia can never again be induced to submit to chloroform or ether.

*No Interference with Peristalsis.*—There is but little, if any, interference with bowel movement. An enema in twenty-four hours usually suffices and even that may not be necessary, for not infrequently there is natural evacuation within the first day, unaided.

*Long Anesthesia.*—The analgesia continues for many hours. This is of distinct advantage in prolonged, tedious operations. To know that the patient is not being carried to the brink of the grave by too much chloroform or ether is an immeasurable comfort to the operator who has to use a long time in his work.

